# Expansion of Dengue Virus Serotype 3 Genetic Diversity and Changing Serotype Composition in Brazil

**DOI:** 10.3390/pathogens15091001

**Published:** 2026-09-21

**Authors:** James Siqueira Pereira, Isabela Carvalho Brcko, Gabriel Montenegro de Campos, Vinicius Carius de Souza, Igor Santana Ribeiro, Mariana Matos Rolle, Brenno Vinicius Martins Henrique, Felicidade Mota Pereira, Arabela Leal e Silva de Mello, Aline de Andrade Freund, Juan Gabriel Carvalho de Araújo, Talita Émile Ribeiro Adelino, Marta Lamounier Moura Vargas Corgozinho, Valnete das Graças Dantas Andrade, Gleissy Adriane Lima Borges, Stephanni Figueiredo da Silva, Elaine Cristina de Oliveira, Glaucilene da Silva Costa, Cicileia Correia da Silva, Mariana de Souza Araujo, Melissa Palmieri, Luiz Carlos Junior Alcantara, Claudia Renata dos Santos Barros, Tiago Souza Salles, Svetoslav Nanev Slavov, Sandra Coccuzzo Sampaio, Maria Carolina Elias, Marta Giovanetti, Alex Ranieri Jeronimo Lima

**Affiliations:** 1 Centro de Vigilância Viral e Avaliação Sorológica (CeVIVAS), Instituto Butantan, Sao Paulo 05503-900, SP, Brazil; jamessiqueirap@gmail.com (J.S.P.); i.brcko.proppg@proppg.butantan.gov.br (I.C.B.); g.campos.esib@esib.butantan.gov.br (G.M.d.C.); vinicius.csouza@fundacaobutantan.org.br (V.C.d.S.); igor.ribeiro.esib@esib.butantan.gov.br (I.S.R.); claudia.barros@fundacaobutantan.org.br (C.R.d.S.B.); tiago.salles@fundacaobutantan.org.br (T.S.S.); svetoslav.slavov@fundacaobutantan.org.br (S.N.S.); sandra.coccuzzo@butantan.gov.br (S.C.S.); carolina.eliassabbaga@butantan.gov.br (M.C.E.); 2 Programa Interunidades de Pós-Graduação em Bioinformática, Universidade de São Paulo, Sao Paulo 05508-000, SP, Brazil; 3 Programa de Pós-Graduação em Doenças Infecciosas e Saúde Global, Faculdade de Medicina, Universidade de São Paulo, Sao Paulo 01246-903, SP, Brazil; 4 Programa de Pós-graduação em Biologia da Relação Patógeno Hospedeiro, Instituto de Ciências Biomédicas, Universidade de São Paulo, Sao Paulo 05508-000, SP, Brazil; 5 Laboratório Central de Saúde Pública do Distrito Federal (LACEN-DF), Brasília 70620-000, DF, Brazil; mariana.roll@saude.df.gov.br (M.M.R.); brenno.henrique@saude.df.gov.br (B.V.M.H.); 6 Laboratório Central de Saúde Pública da Bahia (LACEN-BA), Salvador 41253-190, BA, Brazil; felicidade.pereira@saude.ba.gov.br (F.M.P.);; 7 Laboratório Central de Saúde Pública do Paraná (LACEN-PR), São José dos Pinhais 83060-500, PR, Brazil; alinefreund@sesa.pr.gov.br (A.d.A.F.); juan.araujo@sesa.pr.gov.br (J.G.C.d.A.); 8 Laboratório Central de Saúde Pública de Minas Gerais (LACEN-MG), Fundação Ezequiel Dias, Instituto Otávio Magalhães, Belo Horizonte 30510-010, MG, Brazil; talita.adelino@funed.mg.gov.br (T.É.R.A.); marta.corgozinho@funed.mg.gov.br (M.L.M.V.C.); 9 Laboratório Central de Saúde Pública do Pará (LACEN-PA), Belém 66090-000, PA, Brazil; val.holanda@yahoo.com.br (V.d.G.D.A.); ssyborges@hotmail.com (G.A.L.B.); 10 Laboratório Central de Saúde Pública do Mato Grosso (LACEN-MT), Cuiabá 78043-300, MT, Brazil; stephanni_figueiredo@hotmail.com (S.F.d.S.); ecoelainecristina@gmail.com (E.C.d.O.); 11 Laboratório Central de Saúde Pública de Rondônia (LACEN-RO), Porto Velho 76820-800, RO, Brazil; glaucilene.gsc@gmail.com (G.d.S.C.); cicileiasilva9@gmail.com (C.C.d.S.); 12 Departamento Municipal de Saúde, Secretaria Municipal de Saúde, Prefeitura Municipal de São Paulo, Sao Paulo 01004-000, SP, Brazil; marianasaraujo@prefeitura.sp.gov.br (M.d.S.A.); melissapalmieri@prefeitura.sp.gov.br (M.P.); 13 Instituto René Rachou, Fundação Oswaldo Cruz (Fiocruz), Belo Horizonte 30190-002, MG, Brazil; alcantaraluiz42@gmail.com (L.C.J.A.); giovanetti.marta@gmail.com (M.G.); 14 Climate Amplified Diseases and Epidemics (CLIMADE), Fundação Oswaldo Cruz (Fiocruz), Rio de Janeiro 21040-900, RJ, Brazil; 15 Centro de Desenvolvimento Científico, Instituto Butantan, Sao Paulo 05503-900, SP, Brazil; 16 Department of Science and Technology for Humans and the Environment, Università Campus Bio-Medico di Roma, 00128 Rome, Italy

**Keywords:** dengue virus, DENV-3, serotype composition, phylodynamic

## Abstract

Brazil is the most hyperendemic country for dengue, with historically dominant circulation of DENV-1 and DENV-2. Bayesian phylodynamic analyses of 489 Brazilian DENV-3 genomes, including 84 newly generated sequences, reveal a recent and sustained increase in the effective population size of DENV-3 associated with the recent introduction and expansion of the 3III_B.3.2 lineage. This increase coincides with a sharp rise in the proportion of laboratory-confirmed dengue cases attributed to DENV-3 among records with available serotype information from 2024 onwards and a shift in serotype dynamics in 2025, suggesting a change in serotype distribution and a potential risk of renewed DENV-3 outbreaks, particularly in populations with limited prior exposure to this serotype.

## 1. Introduction

Dengue fever, which can rapidly progress to severe disease and death, is the most important arboviral infection worldwide. It is caused by four distinct serotypes of dengue virus (DENV), a member of the family *Flaviviridae* (genus *Orthoflavivirus*; species *Orthoflavivirus denguei*) [1]. Brazil is the most hyperendemic country for DENV in the world, with circulation of all four serotypes and the highest absolute number of reported cases and deaths. Over the past 15 years, DENV outbreaks in Brazil have been driven primarily by DENV-1 and DENV-2, including the unprecedented 2024 epidemic, with more than 10 million reported cases, surpassing the cumulative total observed from 2011 to 2020 [2,3].

Despite the overall dominance of DENV-1 and DENV-2, a marked increase in the detection of dengue cases associated with DENV-3, a serotype that has circulated at relatively low levels in recent decades, was observed in 2024 across multiple Brazilian states [4]. This pattern has raised concern among public health authorities, particularly given the historically low levels of sustained DENV-3 circulation in Brazil and the possibility of increased population susceptibility to this serotype [5], as well as the expansion of the mosquito vector’s geographic distribution into regions that have historically reported low dengue incidence [6].

Under the most recent DENV lineage nomenclature [7], DENV-3 is classified into five genotypes (I–V). In Brazil, this serotype was introduced in the early twenty-first century, predominantly as genotype 3III [8]. Previous studies have described the re-emergence of DENV-3 and the introduction routes of lineage 3III_B.3.2 in Brazil [4,5,9]. However, whether the recent increase in DENV-3 detection among serotyped dengue cases has been accompanied by changes in viral genetic diversity remains less clearly characterized. To address this gap, we integrated 84 newly generated genomes, extended genomic sampling through 2025, national serotype-specific surveillance data, and Bayesian phylodynamic reconstruction. Here, we investigated whether the recent increase in DENV-3 detection in Brazil was accompanied by changes in viral genetic diversity and DENV-3 inferred effective population size (Ne).

## 2. Materials and Methods

### 2.1. Serotype-Specific Confirmed Dengue Cases

To characterize the temporal distribution of dengue cases associated with each DENV serotype in Brazil, national dengue surveillance data covering the period from January 2014 to December 2025 were retrieved from the Brazilian Notifiable Diseases Information System (SINAN) [10]. Only laboratory-confirmed dengue cases with available serotype information were included, whereas records with unknown or missing serotype information were excluded. Data covering 2014–2024 were accessed on 9 December 2025, and data for 2025 were accessed on 8 January 2026 to allow for annual data consolidation. Serotype-specific data were analyzed descriptively to characterize temporal changes in the relative contribution of DENV-1, DENV-2, DENV-3, and DENV-4 among laboratory-confirmed cases with available serotype information.

### 2.2. Clinical Sample Processing, Sequencing, and Genome Assembly

Viral RNA was extracted using the Extracta Kit AN Viral (Loccus Biotecnologia, Cotia, SP, Brazil) in an automated EXTRACTA 96 system (Loccus Biotecnologia, Cotia, SP, Brazil), following the manufacturer’s instructions. DENV serotype was confirmed using the GeneFinder™ DENV Typing RealAmp Kit (OSang Healthcare Co., Ltd., Anyang, Republic of Korea), a multiplex TaqMan real-time RT-PCR assay targeting the 3′ untranslated region (3′UTR) and capable of detecting all four DENV serotypes.

Samples with cycle threshold (Ct) values below 30 were selected for sequencing. DENV-3 genome amplification was performed using previously described serotype-specific primer sets [5]. Genomic libraries were prepared using the COVIDSeq protocol with the corresponding primer modifications. Libraries were normalized to 4 nM, denatured using 0.2 N NaOH and 400 mM Tris–HCl (pH 8), and sequenced using the NextSeq 500/550 High Output Kit v2.5 (300 cycles) on an Illumina sequencing platform.

Genome assembly was performed using the VIPER (Viral Identification Pipeline for Emergency Response) workflow, publicly available at https://github.com/V-GEN-Lab/viper (accessed on 15 July 2026). Adapter and primer sequences were removed using Cutadapt v2.8 [11], followed by quality filtering with Trimmomatic v0.39 [12]. Filtered reads were mapped against reference genomes representing the four DENV serotypes (DENV-1: NC_001477.1; DENV-2: NC_001474.2; DENV-3: NC_001475.2; DENV-4: NC_002640.1) using BWA-MEM v0.7.17-r1188 [13]. Reads mapped to each reference were quantified from the resulting BAM files using SAMtools v1.12 [14], and the serotype was assigned according to the reference with the highest number of mapped reads.

The BAM file corresponding to the identified serotype was subsequently refined using Pilon v1.24 [15], followed by remapping of the filtered reads and calculation of assembly statistics. Consensus genome sequences were generated using iVar v1.3.1 [16] with a minimum base quality of 20 (-q 20) and minimum depth of 5 (-m 5). Lineage assignment and genome quality assessment were subsequently performed using Nextclade [7,17].

### 2.3. Genomic Dataset Assembly and Phylogenetic Analysis

Publicly available and authorized Brazilian DENV-3 genome sequences were retrieved from the GISAID EpiArbo [18] database (*n* = 308; EPI_SET_260126vu; https://doi.org/10.55876/gis8.260126vu, accessed on 16 December 2025), and from the NCBI GenBank [19] repository, accessed on 10 December 2025 (*n* = 97). Only sequences with ≥80% genome coverage and associated metadata, including at least the year of collection and country-level geographic information, were included in the analyses.

These publicly available sequences were aligned together with the 84 complete DENV-3 genomes generated by partner institutions within the CeVIVAS initiative, available at EPI_SET_260126da. The final dataset (*n* = 489) was aligned using MAFFT v7.520 [20], and alignments were manually inspected using AliView v1.28 [21] to ensure correct codon alignment and overall alignment quality. Sequences were quality-filtered to exclude genomes with excessive ambiguous nucleotides or evidence of sequencing artifacts prior to phylogenetic and phylodynamic inference; no sequences were excluded at this stage.

An initial maximum likelihood (ML) phylogenetic tree was inferred using IQ-TREE2 v2.2.6 [22] (Supplementary Figure S1), applying the GTR+F+I+G4 nucleotide substitution model, as selected by ModelFinder [23] implemented in IQ-TREE2. The robustness of the inferred topology was assessed using 5000 Ultrafast Bootstrap [24] replicates. Metadata for all sequences included in the final dataset are provided in Supplementary Table S1.

### 2.4. Phylodynamic Analysis

Before phylodynamic inference, the temporal signal of the dataset was evaluated using TempEst v1.5.3 [25], based on the correlation between root-to-tip genetic divergence and sampling dates (Supplementary Figure S2). Time-scaled phylogenetic analyses were then performed in BEAST v1.10.4 [26] under a Bayesian framework, using the SRD06 codon-partitioned nucleotide substitution model, an uncorrelated relaxed molecular clock, and a Skygrid coalescent [27] prior with 50 grid points and a time cut-off (TLTP) of 25 years [28]. This coalescent framework was used to infer temporal changes in the effective population size (Ne) and was interpreted as a coalescent-based proxy for viral genetic diversity over time.

Markov chain Monte Carlo (MCMC) analyses were run for 100 million states, with parameters and trees sampled every 10,000 states. Independent MCMC runs were combined and resampled using LogCombiner v1.10.4 [26]. The convergence of the combined runs was then assessed in Tracer v1.7.2 [29] through visual inspection of trace plots and by ensuring ESS values greater than 200 for all relevant parameters.

The posterior distribution of trees was summarized using TreeAnnotator v1.10.4 [26] to generate a maximum clade credibility (MCC) tree. Temporal changes in DENV-3 Ne were inferred from the posterior distribution of trees to characterize changes in viral genetic diversity through time. All downstream analyses and data visualization were performed in R using RStudio v2023.09.1 and the packages tidyverse [30], lubridate [31], ape [32], ggtree [33], ggplot2 [34], readr [35], and dplyr [36].

## 3. Results and Discussion

**Figure 1:**
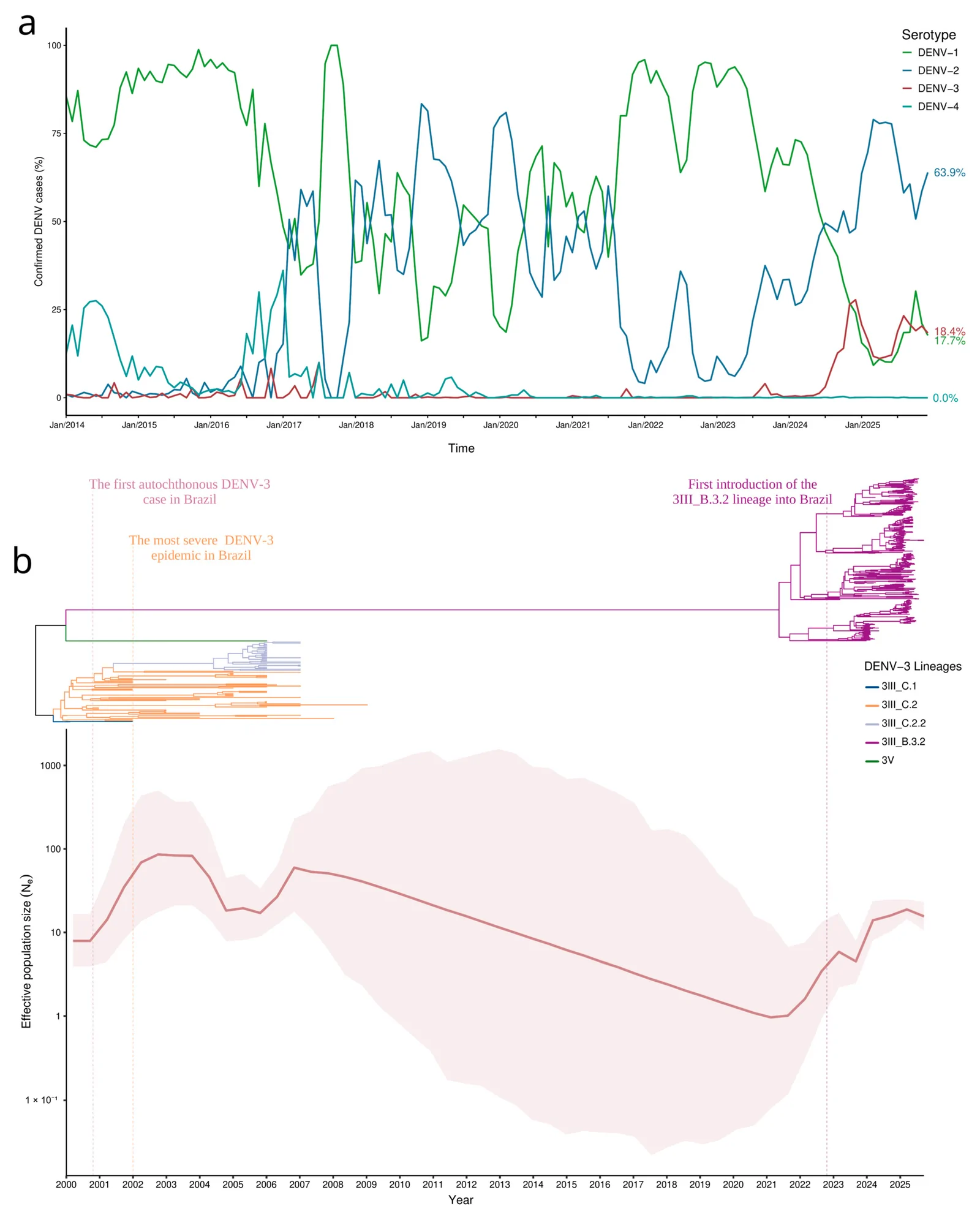
Temporal trends and phylodynamic reconstruction of DENV-3 in Brazil. (**a**) Proportion of laboratory-confirmed dengue cases by serotype from 2014 to 2025. (**b**) Time-scaled phylogeny and Bayesian Skygrid estimate of DENV-3 effective population size (Ne), with shaded areas representing 95% HPD (Highest Posterior Density) intervals and dashed lines indicating key epidemiological events.

Our analysis revealed substantial temporal variation in the inferred DENV-3 Ne in Brazil (Figure 1b). Following the detection of the first autochthonous DENV-3 case in December 2000, marking the introduction of this serotype in the country, viral circulation was primarily dominated by lineage 3III_C.2. During this early phase, inferred Ne increased steadily, preceding and encompassing the largest recorded DENV-3 epidemic in Brazil during 2002–2003, consistent with increasing viral genetic diversity. This increase was followed by a transient decline between approximately 2004 and 2006, compatible with a post-epidemic reduction in circulating viral diversity. Subsequently, from around 2007 to 2008, inferred Ne partially recovered, coinciding with the emergence and co-circulation of lineage 3III_C.2.2 alongside 3III_C.2, suggesting an increase in sampled lineage diversity during this period.

A comparable temporal pattern was observed during the recent period, although in a distinct epidemiological and surveillance context. From 2022 onwards, inferred DENV-3 Ne increased again, temporally coinciding with the introduction and rapid expansion of lineage 3III_B.3.2 in Brazil. The phylogeographic context of this lineage, including evidence for multiple independent introductions into Brazil, was previously investigated by our group [5], while its recent re-emergence in Brazil has also been reported elsewhere [4,9]. This recent increase in inferred Ne, consistent with increasing DENV-3 genetic diversity, preceded the marked rise in the proportion of laboratory-confirmed dengue cases attributed to DENV-3 among records with available serotype information from 2024 onwards. During part of the observed period, DENV-3 surpassed DENV-1 among serotyped confirmed cases, although DENV-2 remained the predominant serotype nationally at the end of the period analyzed (Figure 1a).

Together, these findings indicate that the recent increase in DENV-3 detection in Brazil was accompanied by a phylodynamic signal of increasing viral genetic diversity. Importantly, inferred Ne does not represent a direct estimate of the number of infected individuals and should not be assumed to be proportional to disease incidence. Although the temporal similarity between the early 2000s and the recent period should not be interpreted as evidence that a future epidemic will necessarily occur, it provides a useful context for understanding the epidemiological relevance of the recent expansion of lineage 3III_B.3.2.

This phylodynamic pattern should also be considered alongside broader ecological and immunological conditions that may favor renewed DENV-3 spread in Brazil. The geographic expansion of *Aedes* mosquitoes into previously less affected regions [6,37], together with the prolonged period of limited DENV-3 circulation and multiple recent introduction events into different regions of the country [5], may have created favorable conditions for the establishment and dissemination of this serotype. One plausible, although untested, hypothesis is that population susceptibility to DENV-3 may be relatively high, particularly among younger cohorts with limited prior exposure to this serotype. This scenario is especially relevant because the large dengue epidemic recorded in Brazil in 2024 was predominantly driven by DENV-1 and DENV-2 [38], potentially leaving DENV-3-specific susceptibility unevenly distributed across the population. However, this interpretation should be made cautiously because the present study does not include serological or population immunity data, and the temporal gap in genomic sampling limits direct inference on viral circulation dynamics during the period of low DENV-3 detection.

In Brazil, DENV transmission typically shows a marked seasonal pattern, with most cases occurring between December and June and regional peaks shaped by rainfall, temperature, and vector suitability [39]. In this context, the recent introductions of the 3III_B.3.2 lineage [5], together with the increase in inferred Ne and in the proportion of serotyped confirmed cases attributed to DENV-3, suggest that DENV-3 may become an increasingly important contributor to dengue cases in Brazil during upcoming transmission seasons. If sustained, this scenario could alter the relative contribution of circulating serotypes and increase the need for targeted monitoring of DENV-3-associated outbreaks.

The interpretation of our findings is limited by temporal and geographic gaps in genomic sampling. From 2010 through 2022, the absence of available Brazilian DENV-3 genomes (Supplementary Figure S3) constrained demographic inference, resulting in a long branch leading to the 3III_B.3.2 lineage. This pattern should be interpreted as reflecting substantial unsampled diversity rather than as direct support for continuous local persistence or a specific reintroduction scenario. In addition, the newly generated genomes were geographically concentrated in Minas Gerais and the Federal District, with limited representation from other states. Because heterogeneity in genomic sampling can influence the diversity captured by the dataset and, consequently, the inferred Ne trajectory, part of the recent increase in inferred DENV-3 Ne may reflect differences in genomic sampling and data availability across regions, in addition to underlying epidemiological processes. Therefore, the geographic distribution of available genomes shown in Supplementary Figure S4 should not be interpreted as a direct measure of regional genomic surveillance intensity, as comparable denominators across regions, such as the number of suspected cases, samples tested, or samples selected for sequencing by state, were not available for all data sources.

## 4. Conclusions

Despite these limitations, our study provides evidence that the recent increase in DENV-3 detection among serotyped surveillance records in Brazil was accompanied by a phylodynamic signal of increasing DENV-3 Ne, consistent with an expansion of viral genetic diversity and the circulation of lineage 3III_B.3.2. Together with national serotype-specific surveillance data, these findings indicate a recent shift in the relative contribution of DENV-3 to dengue cases in Brazil, although DENV-2 remained predominant nationally at the end of the period analyzed.

These findings should be interpreted cautiously considering uneven genomic surveillance across the country, the geographic concentration of newly generated genomes, and temporal gaps in available DENV-3 genomic data. Therefore, although the results support the epidemiological relevance of the recent expansion of DENV-3, they should not be taken as evidence of uniform nationwide circulation or as a direct prediction of future epidemic dynamics.

Strengthening genomic surveillance integrated with epidemiological, clinical, immunological, and environmental data will be essential to monitor DENV-3 dynamics and detect changes in circulating serotypes in real time. In this context, tetravalent vaccination remains an important component of dengue preparedness in Brazil; however, its future impact on DENV-3 circulation will depend on vaccine coverage, implementation strategies, target populations, and the evolving serotype composition across the country.

## Data Availability

The newly generated genome sequences have been deposited in GISAID EpiArbo and can be accessed with accession set EPI_SET_260126da (https://doi.org/10.55876/gis8.260126da).

## Supplementary Materials

The following supporting information can be downloaded at: https://www.mdpi.com/article/10.3390/pathogens15091001/s1, Figure S1. Maximum likelihood phylogenetic tree of Brazilian DENV-3 genomes. Tips are colored according to data source: publicly available sequences retrieved from GISAID and GenBank, and new genomes generated in this study through the CeVIVAS initiative. Branch lengths are scaled in substitutions per site; Table S1. Metadata of Brazilian DENV-3 genome sequences used in phylogenetic and phylodynamic analyses, including sample identifiers, collection dates, lineage assignments, and country of origin; Figure S2. Root-to-tip regression analysis of the Brazilian DENV-3 dataset. The genetic divergence (root-to-tip distance) is plotted against sampling dates to assess the temporal signal. The strong linear relationship (correlation coefficient = 0.98; R^2^ = 0.96) indicates a robust molecular clock signal, supporting the suitability of the dataset for time-scaled phylogenetic and phylodynamic analyses; Figure S3. Temporal distribution of DENV-3 genomes sequenced in Brazil between 2001 and 2025. The line plot shows the annual number of genomes available, with points representing the total number for each year. Years with no available genomic data are shown with zero counts, highlighting prolonged periods of limited genomic surveillance.; Figure S4. Geographic distribution of Brazilian DENV-3 genomes included in this study. (a) Brazilian states represented in the genomic dataset according to data source. “Both sources” indicates states with both newly generated genomes and publicly available sequences, “This study only” indicates states represented only by newly generated genomes, and “Public data only” indicates states represented only by sequences retrieved from public databases. (b) Number of Brazilian DENV-3 genomes by state, data source, and lineage among sequences included in the dataset. Sequences without state-level metadata are shown separately as “No state metadata” in panel b and were not represented in the map.

## Abbreviations

The following abbreviations are used in this manuscript:

3′UTR	3′ untranslated region
BAM	Binary Alignment/Map
BEAST	Bayesian Evolutionary Analysis by Sampling Trees
CAPES	Coordenação de Aperfeiçoamento de Pessoal de Nível Superior
CeVIVAS	Centro de Vigilância Viral e Avaliação Sorológica
CLIMADE	Climate Amplified Diseases and Epidemics
CNPq	National Council for Scientific and Technological Development
Ct	Cycle threshold
DENV	Dengue virus
DENV-1	Dengue virus serotype 1
DENV-2	Dengue virus serotype 2
DENV-3	Dengue virus serotype 3
DENV-4	Dengue virus serotype 4
ESS	Effective sample size
FAPESP	Fundação de Amparo à Pesquisa do Estado de São Paulo
Fiocruz	Fundação Oswaldo Cruz
GISAID	Global Initiative on Sharing All Influenza Data
HPD	Highest Posterior Density
IQ-TREE2	IQ-TREE version 2
LACEN	Laboratório Central de Saúde Pública
LACEN-BA	Laboratório Central de Saúde Pública da Bahia
LACEN-DF	Laboratório Central de Saúde Pública do Distrito Federal
LACEN-MG	Laboratório Central de Saúde Pública de Minas Gerais
LACEN-MT	Laboratório Central de Saúde Pública do Mato Grosso
LACEN-PA	Laboratório Central de Saúde Pública do Pará
LACEN-PR	Laboratório Central de Saúde Pública do Paraná
LACEN-RO	Laboratório Central de Saúde Pública de Rondônia
LEDM	Laboratório Estratégico de Diagnóstico Molecular
MAFFT	Multiple Alignment using Fast Fourier Transform
MCC	Maximum clade credibility
MCMC	Markov chain Monte Carlo
ML	Maximum likelihood
NBBC	Center for Bioinformatics and Computational Biology
NCBI	National Center for Biotechnology Information
Ne	Effective population size
RT-PCR	Reverse transcription polymerase chain reaction
SINAN	Brazilian Notifiable Diseases Information System
SRD06	Shapiro–Rambaut–Drummond 2006 codon-partitioned nucleotide substitution model
TLTP	Time to the Last Transition Point
UFBoot	Ultrafast Bootstrap
USP	Universidade de São Paulo
VIPER	Viral Identification Pipeline for Emergency Response
