# Supplementary material for: Expansion of Dengue Virus Serotype 3 Genetic Diversity and Changing Serotype Composition in Brazil
Source: Pathogens. 2026 Sep 21;15(9):1001. doi: 10.3390/pathogens15091001 (PMC13609987; doi:10.3390/pathogens15091001)
Supplement: Supplementary file 1 [file pathogens-15-01001-s001.zip › pathogens-4487624-supplementary.pdf]

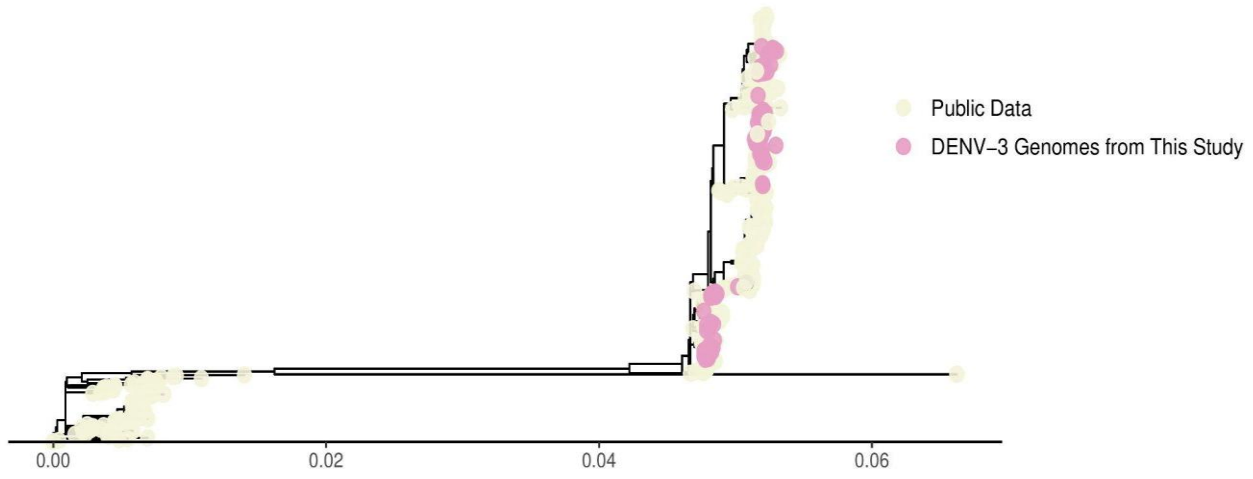

Supplementary Figure S1. Maximum likelihood phylogenetic tree of Brazilian DENV-3 genomes. Tips are colored according to data source: publicly available sequences retrieved from GISAID and GenBank, and new genomes generated in this study through the CeVIVAS initiative. Branch lengths are scaled in substitutions per site.

Supplementary Table S1. Metadata of Brazilian DENV-3 genome sequences used in phylogenetic and phylodynamic analyses, including sample identifiers, collection dates, lineage assignments, and country of origin.

	ID	Collection date	Lineage	Country		ID	Collection date	Lineage	Country
**1**	**EPI_ISL_19897973**	2024-04-30	3III_B.3.2	Brazil	**246**	EPI_ISL_19908995	2025-03-22	3III_B.3.2	Brazil
**2**	**EPI_ISL_19897876**	2025-02-11	3III_B.3.2	Brazil	**247**	EPI_ISL_19908996	2025-02-03	3III_B.3.2	Brazil
**3**	**EPI_ISL_19897878**	2025-01-05	3III_B.3.2	Brazil	**248**	EPI_ISL_19908997	2025-02-06	3III_B.3.2	Brazil
**4**	**EPI_ISL_19897889**	2025-02-17	3III_B.3.2	Brazil	**249**	EPI_ISL_19908998	2025-02-07	3III_B.3.2	Brazil
**5**	**EPI_ISL_19897896**	2025-01-24	3III_B.3.2	Brazil	**250**	EPI_ISL_19908999	2025-02-25	3III_B.3.2	Brazil
**6**	**EPI_ISL_19897897**	2025-02-06	3III_B.3.2	Brazil	**251**	EPI_ISL_20123511	2025-03-31	3III_B.3.2	Brazil
**7**	**EPI_ISL_19897901**	2025-02-03	3III_B.3.2	Brazil	**252**	EPI_ISL_20123514	2025-06-04	3III_B.3.2	Brazil
**8**	**EPI_ISL_19897902**	2025-02-17	3III_B.3.2	Brazil	**253**	EPI_ISL_20123521	2025-06-08	3III_B.3.2	Brazil
**9**	**EPI_ISL_19897903**	2025-02-07	3III_B.3.2	Brazil	**254**	EPI_ISL_20123522	2025-06-12	3III_B.3.2	Brazil
**10**	**EPI_ISL_19897904**	2025-02-07	3III_B.3.2	Brazil	**255**	EPI_ISL_20123523	2025-06-20	3III_B.3.2	Brazil
**11**	**EPI_ISL_19897908**	2025-02-03	3III_B.3.2	Brazil	**256**	EPI_ISL_20123527	2025-06-15	3III_B.3.2	Brazil
**12**	**EPI_ISL_19897910**	2025-02-03	3III_B.3.2	Brazil	**257**	EPI_ISL_20123541	2025-06-06	3III_B.3.2	Brazil
**13**	**EPI_ISL_19897911**	2025-02-07	3III_B.3.2	Brazil	**258**	EPI_ISL_20123544	2025-05-25	3III_B.3.2	Brazil
**14**	**EPI_ISL_19897912**	2025-01-05	3III_B.3.2	Brazil	**259**	EPI_ISL_20123547	2025-03-27	3III_B.3.2	Brazil
**15**	**EPI_ISL_19897913**	2025-02-26	3III_B.3.2	Brazil	**260**	EPI_ISL_20123556	2025-03-31	3III_B.3.2	Brazil
**16**	**EPI_ISL_19897916**	2025-02-05	3III_B.3.2	Brazil	**261**	EPI_ISL_20123562	2025-05-27	3III_B.3.2	Brazil
**17**	**EPI_ISL_19897920**	2025-01-28	3III_B.3.2	Brazil	**262**	EPI_ISL_20123564	2025-05-24	3III_B.3.2	Brazil
**18**	**EPI_ISL_19897924**	2025-02-05	3III_B.3.2	Brazil	**263**	EPI_ISL_20123567	2025-05-27	3III_B.3.2	Brazil
**19**	**EPI_ISL_19897926**	2025-02-06	3III_B.3.2	Brazil	**264**	EPI_ISL_20125207	2025-05-21	3III_B.3.2	Brazil
**20**	**EPI_ISL_19897927**	2025-02-23	3III_B.3.2	Brazil	**265**	EPI_ISL_20125208	2025-05-26	3III_B.3.2	Brazil
**21**	**EPI_ISL_19897928**	2025-02-26	3III_B.3.2	Brazil	**266**	EPI_ISL_20125209	2025-05-26	3III_B.3.2	Brazil
**22**	**EPI_ISL_19897930**	2025-01-31	3III_B.3.2	Brazil	**267**	EPI_ISL_20125210	2025-05-23	3III_B.3.2	Brazil
**23**	**EPI_ISL_19897934**	2025-01-21	3III_B.3.2	Brazil	**268**	EPI_ISL_20125211	2025-05-31	3III_B.3.2	Brazil

	ID	Collection date	Lineage	Country		ID	Collection date	Lineage	Country
**24**	**EPI_ISL_19897935**	2025-02-06	3III_B.3.2	Brazil	**269**	EPI_ISL_20125212	2025-06-02	3III_B.3.2	Brazil
**25**	**EPI_ISL_19897938**	2025-02-07	3III_B.3.2	Brazil	**270**	EPI_ISL_20125213	2025-06-01	3III_B.3.2	Brazil
**26**	**EPI_ISL_19897942**	2025-01-20	3III_B.3.2	Brazil	**271**	EPI_ISL_20125214	2025-06-02	3III_B.3.2	Brazil
**27**	**EPI_ISL_19897947**	2025-01-28	3III_B.3.2	Brazil	**272**	EPI_ISL_20125215	2025-06-05	3III_B.3.2	Brazil
**28**	**EPI_ISL_19897948**	2025-02-25	3III_B.3.2	Brazil	**273**	EPI_ISL_20125216	2025-06-09	3III_B.3.2	Brazil
**29**	**EPI_ISL_19897950**	2025-01-21	3III_B.3.2	Brazil	**274**	EPI_ISL_20125217	2025-06-08	3III_B.3.2	Brazil
**30**	**EPI_ISL_19897951**	2025-02-03	3III_B.3.2	Brazil	**275**	EPI_ISL_20125218	2025-06-10	3III_B.3.2	Brazil
**31**	**EPI_ISL_19897953**	2025-02-10	3III_B.3.2	Brazil	**276**	EPI_ISL_20125219	2025-06-22	3III_B.3.2	Brazil
**32**	**EPI_ISL_19897954**	2025-02-04	3III_B.3.2	Brazil	**277**	EPI_ISL_20125220	2025-06-24	3III_B.3.2	Brazil
**33**	**EPI_ISL_19897955**	2025-01-21	3III_B.3.2	Brazil	**278**	EPI_ISL_20125221	2025-04-28	3III_B.3.2	Brazil
**34**	**EPI_ISL_19897956**	2025-01-23	3III_B.3.2	Brazil	**279**	EPI_ISL_20125222	2025-04-25	3III_B.3.2	Brazil
**35**	**EPI_ISL_19897957**	2025-02-04	3III_B.3.2	Brazil	**280**	EPI_ISL_20125223	2025-04-25	3III_B.3.2	Brazil
**36**	**EPI_ISL_19897958**	2025-02-25	3III_B.3.2	Brazil	**281**	EPI_ISL_20125224	2025-04-25	3III_B.3.2	Brazil
**37**	**EPI_ISL_19897959**	2025-02-07	3III_B.3.2	Brazil	**282**	EPI_ISL_20125225	2025-04-27	3III_B.3.2	Brazil
**38**	**EPI_ISL_19897960**	2025-02-24	3III_B.3.2	Brazil	**283**	EPI_ISL_20125226	2025-04-25	3III_B.3.2	Brazil
**39**	**EPI_ISL_19897961**	2025-03-05	3III_B.3.2	Brazil	**284**	EPI_ISL_20125227	2025-04-24	3III_B.3.2	Brazil
**40**	**EPI_ISL_19897962**	2025-02-06	3III_B.3.2	Brazil	**285**	EPI_ISL_20125228	2025-04-29	3III_B.3.2	Brazil
**41**	**EPI_ISL_19897963**	2025-02-06	3III_B.3.2	Brazil	**286**	EPI_ISL_20125229	2025-04-30	3III_B.3.2	Brazil
**42**	**EPI_ISL_19897964**	2025-02-13	3III_B.3.2	Brazil	**287**	EPI_ISL_20125230	2025-04-30	3III_B.3.2	Brazil
**43**	**EPI_ISL_19897965**	2025-02-18	3III_B.3.2	Brazil	**288**	EPI_ISL_20125231	2025-04-23	3III_B.3.2	Brazil
**44**	**EPI_ISL_20336941**	2025-02-11	3III_B.3.2	Brazil	**289**	EPI_ISL_20125232	2025-05-04	3III_B.3.2	Brazil
**45**	**EPI_ISL_20336942**	2025-01-31	3III_B.3.2	Brazil	**290**	EPI_ISL_20125233	2025-04-29	3III_B.3.2	Brazil
**46**	**EPI_ISL_20336943**	2025-03-04	3III_B.3.2	Brazil	**291**	EPI_ISL_20125234	2025-05-05	3III_B.3.2	Brazil
**47**	**EPI_ISL_20336944**	2025-02-04	3III_B.3.2	Brazil	**292**	EPI_ISL_20125235	2025-04-30	3III_B.3.2	Brazil
**48**	**EPI_ISL_20336945**	2025-02-13	3III_B.3.2	Brazil	**293**	EPI_ISL_20125236	2025-05-06	3III_B.3.2	Brazil
**49**	**EPI_ISL_20336946**	2025-01-31	3III_B.3.2	Brazil	**294**	EPI_ISL_20125237	2025-05-07	3III_B.3.2	Brazil

	ID	Collection date	Lineage	Country		ID	Collection date	Lineage	Country
**50**	**EPI_ISL_20336947**	2025-02-12	3III_B.3.2	Brazil	**295**	EPI_ISL_20125238	2025-04-22	3III_B.3.2	Brazil
**51**	**EPI_ISL_20336948**	2025-01-22	3III_B.3.2	Brazil	**296**	EPI_ISL_20125239	2025-05-01	3III_B.3.2	Brazil
**52**	**EPI_ISL_20336949**	2025-02-14	3III_B.3.2	Brazil	**297**	EPI_ISL_20125240	2025-05-05	3III_B.3.2	Brazil
**53**	**EPI_ISL_20336950**	2025-02-05	3III_B.3.2	Brazil	**298**	EPI_ISL_20125241	2025-05-06	3III_B.3.2	Brazil
**54**	**EPI_ISL_20336951**	2025-01-30	3III_B.3.2	Brazil	**299**	EPI_ISL_20125242	2025-05-05	3III_B.3.2	Brazil
**55**	**EPI_ISL_20336952**	2025-02-05	3III_B.3.2	Brazil	**300**	EPI_ISL_20125243	2025-05-06	3III_B.3.2	Brazil
**56**	**EPI_ISL_19884841**	2025-02-17	3III_B.3.2	Brazil	**301**	EPI_ISL_20125244	2025-05-08	3III_B.3.2	Brazil
**57**	**EPI_ISL_19884840**	2025-02-03	3III_B.3.2	Brazil	**302**	EPI_ISL_20125245	2025-04-05	3III_B.3.2	Brazil
**58**	**EPI_ISL_19884839**	2025-05-16	3III_B.3.2	Brazil	**303**	EPI_ISL_20125246	2025-04-05	3III_B.3.2	Brazil
**59**	**EPI_ISL_19884838**	2025-05-12	3III_B.3.2	Brazil	**304**	EPI_ISL_20125247	2025-04-08	3III_B.3.2	Brazil
**60**	**EPI_ISL_19884836**	2025-05-14	3III_B.3.2	Brazil	**305**	EPI_ISL_20125248	2025-04-08	3III_B.3.2	Brazil
**61**	**EPI_ISL_19884835**	2025-05-10	3III_B.3.2	Brazil	**306**	EPI_ISL_20125249	2025-04-12	3III_B.3.2	Brazil
**62**	**EPI_ISL_19884832**	2025-04-30	3III_B.3.2	Brazil	**307**	EPI_ISL_20125250	2025-04-15	3III_B.3.2	Brazil
**63**	**EPI_ISL_19884831**	2025-04-26	3III_B.3.2	Brazil	**308**	EPI_ISL_20125251	2025-04-16	3III_B.3.2	Brazil
**64**	**EPI_ISL_19884830**	2025-04-26	3III_B.3.2	Brazil	**309**	EPI_ISL_20125252	2025-04-14	3III_B.3.2	Brazil
**65**	**EPI_ISL_19884829**	2025-04-23	3III_B.3.2	Brazil	**310**	EPI_ISL_20125253	2025-04-24	3III_B.3.2	Brazil
**66**	**EPI_ISL_19884827**	2025-04-15	3III_B.3.2	Brazil	**311**	EPI_ISL_20125254	2025-04-25	3III_B.3.2	Brazil
**67**	**EPI_ISL_19884826**	2025-04-14	3III_B.3.2	Brazil	**312**	EPI_ISL_20132200	2025-05-23	3III_B.3.2	Brazil
**68**	**EPI_ISL_19884825**	2025-04-07	3III_B.3.2	Brazil	**313**	EPI_ISL_20132201	2025-05-29	3III_B.3.2	Brazil
**69**	**EPI_ISL_19884823**	2025-04-03	3III_B.3.2	Brazil	**314**	EPI_ISL_20132202	2025-05-29	3III_B.3.2	Brazil
**70**	**EPI_ISL_19884822**	2025-04-03	3III_B.3.2	Brazil	**315**	EPI_ISL_20132203	2025-06-08	3III_B.3.2	Brazil
**71**	**EPI_ISL_19884821**	2025-03-29	3III_B.3.2	Brazil	**316**	EPI_ISL_20132204	2025-05-13	3III_B.3.2	Brazil
**72**	**EPI_ISL_19884820**	2025-03-12	3III_B.3.2	Brazil	**317**	EPI_ISL_20132205	2025-05-14	3III_B.3.2	Brazil
**73**	**EPI_ISL_19884819**	2025-03-08	3III_B.3.2	Brazil	**318**	EPI_ISL_20132206	2025-05-13	3III_B.3.2	Brazil
**74**	**EPI_ISL_19884818**	2025-03-07	3III_B.3.2	Brazil	**319**	EPI_ISL_20132207	2025-06-08	3III_B.3.2	Brazil
**75**	**EPI_ISL_19884817**	2025-03-04	3III_B.3.2	Brazil	**320**	EPI_ISL_20155574	2025-07-16	3III_B.3.2	Brazil

	ID	Collection date	Lineage	Country		ID	Collection date	Lineage	Country
**76**	**EPI_ISL_19884816**	2025-03-03	3III_B.3.2	Brazil	**321**	EPI_ISL_20173932	2025-05-13	3III_B.3.2	Brazil
**77**	**EPI_ISL_19884837**	2025-05-13	3III_B.3.2	Brazil	**322**	EPI_ISL_20173933	2025-05-13	3III_B.3.2	Brazil
**78**	**EPI_ISL_19884834**	2025-04-28	3III_B.3.2	Brazil	**323**	EPI_ISL_20173934	2025-05-13	3III_B.3.2	Brazil
**79**	**EPI_ISL_19884828**	2025-04-22	3III_B.3.2	Brazil	**324**	EPI_ISL_20173935	2025-05-12	3III_B.3.2	Brazil
**80**	**EPI_ISL_19884824**	2025-04-03	3III_B.3.2	Brazil	**325**	EPI_ISL_20173936	2025-05-12	3III_B.3.2	Brazil
**81**	**EPI_ISL_20336953**	2025-06-03	3III_B.3.2	Brazil	**326**	EPI_ISL_20173937	2025-05-13	3III_B.3.2	Brazil
**82**	**EPI_ISL_20336954**	2025-03-06	3III_B.3.2	Brazil	**327**	EPI_ISL_20173939	2025-05-15	3III_B.3.2	Brazil
**83**	**EPI_ISL_20257062**	2025-09-18	3III_B.3.2	Brazil	**328**	EPI_ISL_20173940	2025-05-17	3III_B.3.2	Brazil
**84**	**EPI_ISL_20257063**	2025-09-16	3III_B.3.2	Brazil	**329**	EPI_ISL_20173941	2025-05-18	3III_B.3.2	Brazil
**85**	EPI_ISL_19446001	2024-05-14	3III_B.3.2	Brazil	**330**	EPI_ISL_20173942	2025-05-15	3III_B.3.2	Brazil
**86**	EPI_ISL_19796938	2024-03-26	3III_B.3.2	Brazil	**331**	EPI_ISL_20173943	2025-05-19	3III_B.3.2	Brazil
**87**	EPI_ISL_19796937	2024-03-06	3III_B.3.2	Brazil	**332**	EPI_ISL_20173944	2025-05-21	3III_B.3.2	Brazil
**88**	EPI_ISL_19796936	2024-03-04	3III_B.3.2	Brazil	**333**	EPI_ISL_20173945	2025-05-23	3III_B.3.2	Brazil
**89**	EPI_ISL_19796935	2024-01-24	3III_B.3.2	Brazil	**334**	EPI_ISL_20173949	2025-05-21	3III_B.3.2	Brazil
**90**	EPI_ISL_19742828	2024-08-21	3III_B.3.2	Brazil	**335**	EPI_ISL_20173950	2025-05-21	3III_B.3.2	Brazil
**91**	EPI_ISL_19735142	2024-10-25	3III_B.3.2	Brazil	**336**	EPI_ISL_20173951	2025-05-21	3III_B.3.2	Brazil
**92**	EPI_ISL_18542103	2023-11-09	3III_B.3.2	Brazil	**337**	EPI_ISL_20173952	2025-05-21	3III_B.3.2	Brazil
**93**	EPI_ISL_18699410	2023-11-21	3III_B.3.2	Brazil	**338**	EPI_ISL_20173953	2025-05-21	3III_B.3.2	Brazil
**94**	EPI_ISL_18755964	2023-12-25	3III_B.3.2	Brazil	**339**	EPI_ISL_20173954	2025-05-21	3III_B.3.2	Brazil
**95**	EPI_ISL_18871003	2023-11-28	3III_B.3.2	Brazil	**340**	EPI_ISL_20173955	2025-05-21	3III_B.3.2	Brazil
**96**	EPI_ISL_18871004	2023-11-28	3III_B.3.2	Brazil	**341**	EPI_ISL_20173956	2025-05-16	3III_B.3.2	Brazil
**97**	EPI_ISL_18871005	2023-12-04	3III_B.3.2	Brazil	**342**	EPI_ISL_20173957	2025-05-16	3III_B.3.2	Brazil
**98**	EPI_ISL_18871006	2023-12-29	3III_B.3.2	Brazil	**343**	EPI_ISL_20173958	2025-05-19	3III_B.3.2	Brazil
**99**	EPI_ISL_18871007	2023-12-28	3III_B.3.2	Brazil	**344**	EPI_ISL_20173959	2025-05-19	3III_B.3.2	Brazil
**100**	EPI_ISL_18953953	2024-01-18	3III_B.3.2	Brazil	**345**	EPI_ISL_20173960	2025-05-16	3III_B.3.2	Brazil
**101**	EPI_ISL_18953954	2024-01-19	3III_B.3.2	Brazil	**346**	EPI_ISL_20173961	2025-05-16	3III_B.3.2	Brazil

	ID	Collection date	Lineage	Country		ID	Collection date	Lineage	Country
**102**	EPI_ISL_18953955	2024-01-23	3III_B.3.2	Brazil	**347**	EPI_ISL_20173962	2025-05-19	3III_B.3.2	Brazil
**103**	EPI_ISL_18953956	2024-01-24	3III_B.3.2	Brazil	**348**	EPI_ISL_20173963	2025-05-14	3III_B.3.2	Brazil
**104**	EPI_ISL_18953957	2024-01-24	3III_B.3.2	Brazil	**349**	EPI_ISL_20173964	2025-04-15	3III_B.3.2	Brazil
**105**	EPI_ISL_18953958	2024-01-25	3III_B.3.2	Brazil	**350**	EPI_ISL_20173965	2025-04-14	3III_B.3.2	Brazil
**106**	EPI_ISL_18953959	2024-01-25	3III_B.3.2	Brazil	**351**	EPI_ISL_20173966	2025-04-03	3III_B.3.2	Brazil
**107**	EPI_ISL_18953960	2024-01-29	3III_B.3.2	Brazil	**352**	EPI_ISL_20173967	2025-04-04	3III_B.3.2	Brazil
**108**	EPI_ISL_19075352	2024-03-15	3III_B.3.2	Brazil	**353**	EPI_ISL_20173968	2025-05-19	3III_B.3.2	Brazil
**109**	EPI_ISL_19076631	2024-02-02	3III_B.3.2	Brazil	**354**	EPI_ISL_20173969	2025-05-19	3III_B.3.2	Brazil
**110**	EPI_ISL_19076632	2024-02-05	3III_B.3.2	Brazil	**355**	EPI_ISL_20173970	2025-05-16	3III_B.3.2	Brazil
**111**	EPI_ISL_19076633	2024-02-02	3III_B.3.2	Brazil	**356**	EPI_ISL_20173971	2025-05-13	3III_B.3.2	Brazil
**112**	EPI_ISL_19076634	2024-02-06	3III_B.3.2	Brazil	**357**	EPI_ISL_20173973	2025-05-22	3III_B.3.2	Brazil
**113**	EPI_ISL_19076635	2024-02-06	3III_B.3.2	Brazil	**358**	EPI_ISL_20173974	2025-05-21	3III_B.3.2	Brazil
**114**	EPI_ISL_19076636	2024-02-06	3III_B.3.2	Brazil	**359**	EPI_ISL_20183283	2025-04-09	3III_B.3.2	Brazil
**115**	EPI_ISL_19076637	2024-02-07	3III_B.3.2	Brazil	**360**	EPI_ISL_20183284	2025-04-15	3III_B.3.2	Brazil
**116**	EPI_ISL_19076638	2024-02-08	3III_B.3.2	Brazil	**361**	EPI_ISL_20183285	2025-04-29	3III_B.3.2	Brazil
**117**	EPI_ISL_19076639	2024-02-08	3III_B.3.2	Brazil	**362**	EPI_ISL_20183286	2025-04-29	3III_B.3.2	Brazil
**118**	EPI_ISL_19076640	2024-02-07	3III_B.3.2	Brazil	**363**	EPI_ISL_20183287	2025-05-07	3III_B.3.2	Brazil
**119**	EPI_ISL_19076641	2024-02-09	3III_B.3.2	Brazil	**364**	EPI_ISL_20183288	2025-05-15	3III_B.3.2	Brazil
**120**	EPI_ISL_19076642	2024-01-05	3III_B.3.2	Brazil	**365**	EPI_ISL_20183289	2025-05-15	3III_B.3.2	Brazil
**121**	EPI_ISL_19179567	2024-03-22	3III_B.3.2	Brazil	**366**	EPI_ISL_20183290	2025-05-15	3III_B.3.2	Brazil
**122**	EPI_ISL_19179568	2024-03-13	3III_B.3.2	Brazil	**367**	EPI_ISL_20183291	2025-06-05	3III_B.3.2	Brazil
**123**	EPI_ISL_19179569	2024-04-06	3III_B.3.2	Brazil	**368**	EPI_ISL_20183292	2025-07-01	3III_B.3.2	Brazil
**124**	EPI_ISL_19459672	2024-02-05	3III_B.3.2	Brazil	**369**	EPI_ISL_20183293	2025-07-03	3III_B.3.2	Brazil
**125**	EPI_ISL_19459673	2024-03-26	3III_B.3.2	Brazil	**370**	EPI_ISL_20183689	2025-07-13	3III_B.3.2	Brazil
**126**	EPI_ISL_19459674	2024-03-07	3III_B.3.2	Brazil	**371**	EPI_ISL_20183690	2025-07-12	3III_B.3.2	Brazil
**127**	EPI_ISL_19459675	2024-02-05	3III_B.3.2	Brazil	**372**	EPI_ISL_20183691	2025-07-06	3III_B.3.2	Brazil

	ID	Collection date	Lineage	Country		ID	Collection date	Lineage	Country
**128**	EPI_ISL_19459676	2024-01-29	3III_B.3.2	Brazil	**373**	EPI_ISL_20183692	2025-06-30	3III_B.3.2	Brazil
**129**	EPI_ISL_19459677	2024-01-29	3III_B.3.2	Brazil	**374**	EPI_ISL_20183693	2025-06-29	3III_B.3.2	Brazil
**130**	EPI_ISL_19459678	2024-02-02	3III_B.3.2	Brazil	**375**	EPI_ISL_20183695	2025-07-24	3III_B.3.2	Brazil
**131**	EPI_ISL_19459679	2024-01-26	3III_B.3.2	Brazil	**376**	EPI_ISL_20183696	2025-07-23	3III_B.3.2	Brazil
**132**	EPI_ISL_19459680	2024-01-29	3III_B.3.2	Brazil	**377**	EPI_ISL_20183697	2025-07-04	3III_B.3.2	Brazil
**133**	EPI_ISL_19459681	2024-01-26	3III_B.3.2	Brazil	**378**	EPI_ISL_20183698	2025-07-21	3III_B.3.2	Brazil
**134**	EPI_ISL_19459682	2024-01-29	3III_B.3.2	Brazil	**379**	EPI_ISL_20193196	2025-02-25	3III_B.3.2	Brazil
**135**	EPI_ISL_19459683	2024-01-26	3III_B.3.2	Brazil	**380**	EPI_ISL_20193198	2025-03-04	3III_B.3.2	Brazil
**136**	EPI_ISL_19459684	2024-03-07	3III_B.3.2	Brazil	**381**	EPI_ISL_20193202	2025-01-24	3III_B.3.2	Brazil
**137**	EPI_ISL_19459685	2024-01-26	3III_B.3.2	Brazil	**382**	EPI_ISL_20193204	2025-02-05	3III_B.3.2	Brazil
**138**	EPI_ISL_19459686	2024-02-14	3III_B.3.2	Brazil	**383**	EPI_ISL_20193208	2025-01-23	3III_B.3.2	Brazil
**139**	EPI_ISL_19459687	2024-03-27	3III_B.3.2	Brazil	**384**	EPI_ISL_20193209	2025-01-31	3III_B.3.2	Brazil
**140**	EPI_ISL_19459688	2024-02-06	3III_B.3.2	Brazil	**385**	EPI_ISL_20193211	2025-01-23	3III_B.3.2	Brazil
**141**	EPI_ISL_19459689	2024-02-06	3III_B.3.2	Brazil	**386**	EPI_ISL_20193214	2025-02-12	3III_B.3.2	Brazil
**142**	EPI_ISL_19459690	2024-05-09	3III_B.3.2	Brazil	**387**	EPI_ISL_20193215	2025-02-05	3III_B.3.2	Brazil
**143**	EPI_ISL_19459691	2024-01-29	3III_B.3.2	Brazil	**388**	EPI_ISL_20193216	2025-01-05	3III_B.3.2	Brazil
**144**	EPI_ISL_19459692	2024-01-30	3III_B.3.2	Brazil	**389**	EPI_ISL_20193217	2025-02-07	3III_B.3.2	Brazil
**145**	EPI_ISL_19459693	2024-01-30	3III_B.3.2	Brazil	**390**	EPI_ISL_20193218	2025-02-07	3III_B.3.2	Brazil
**146**	EPI_ISL_19520623	2024-06-13	3III_B.3.2	Brazil	**391**	EPI_ISL_20193219	2025-02-17	3III_B.3.2	Brazil
**147**	EPI_ISL_19520624	2024-06-13	3III_B.3.2	Brazil	**392**	EPI_ISL_20193220	2025-02-07	3III_B.3.2	Brazil
**148**	EPI_ISL_19520626	2024-04-01	3III_B.3.2	Brazil	**393**	FJ850079.1	2003-01-01	3III_C.1	Brazil
**149**	EPI_ISL_19609374	2024-03-25	3III_B.3.2	Brazil	**394**	FJ850080.1	2003-01-01	3III_C.2	Brazil
**150**	EPI_ISL_19609375	2024-04-03	3III_B.3.2	Brazil	**395**	FJ850083.1	2004-01-01	3III_C.2	Brazil
**151**	EPI_ISL_19609376	2024-05-12	3III_B.3.2	Brazil	**396**	FJ850086.1	2005-01-01	3III_C.2	Brazil
**152**	EPI_ISL_19609377	2024-10-16	3III_B.3.2	Brazil	**397**	FJ850089.1	2006-01-01	3III_C.2	Brazil
**153**	EPI_ISL_19704148	2024-12-15	3III_B.3.2	Brazil	**398**	FJ850092.1	2007-01-01	3III_C.2	Brazil

	ID	Collection date	Lineage	Country		ID	Collection date	Lineage	Country
**154**	EPI_ISL_19750068	2024-12-16	3III_B.3.2	Brazil	**399**	FJ850094.1	2008-01-01	3III_C.2	Brazil
**155**	EPI_ISL_19750069	2024-12-13	3III_B.3.2	Brazil	**400**	FJ898446.1	2001-01-01	3III_C.2	Brazil
**156**	EPI_ISL_19750070	2024-12-30	3III_B.3.2	Brazil	**401**	FJ898447.1	2003-01-01	3III_C.2	Brazil
**157**	EPI_ISL_19750071	2025-01-07	3III_B.3.2	Brazil	**402**	FJ913015.1	2001-01-01	3III_C.2	Brazil
**158**	EPI_ISL_19750072	2025-01-08	3III_B.3.2	Brazil	**403**	GQ868546.1	2006-01-01	3III_C.2.2	Brazil
**159**	EPI_ISL_19750073	2025-01-15	3III_B.3.2	Brazil	**404**	GQ868547.1	2006-01-01	3III_C.2.2	Brazil
**160**	EPI_ISL_19750074	2025-01-14	3III_B.3.2	Brazil	**405**	GQ868548.1	2006-01-01	3III_C.2.2	Brazil
**161**	EPI_ISL_19780844	2025-02-26	3III_B.3.2	Brazil	**406**	GU131844.1	2006-01-01	3III_C.2	Brazil
**162**	EPI_ISL_19784619	2024-11-18	3III_B.3.2	Brazil	**407**	GU131845.1	2006-01-01	3III_C.2.2	Brazil
**163**	EPI_ISL_19784620	2024-09-13	3III_B.3.2	Brazil	**408**	GU131846.1	2006-01-01	3III_C.2.2	Brazil
**164**	EPI_ISL_19797802	2025-02-05	3III_B.3.2	Brazil	**409**	GU131847.1	2006-01-01	3III_C.2.2	Brazil
**165**	EPI_ISL_19797803	2025-02-10	3III_B.3.2	Brazil	**410**	GU131848.1	2006-01-01	3III_C.2.2	Brazil
**166**	EPI_ISL_19797804	2025-02-06	3III_B.3.2	Brazil	**411**	GU131849.1	2006-01-01	3III_C.2.2	Brazil
**167**	EPI_ISL_19797807	2025-02-12	3III_B.3.2	Brazil	**412**	GU131850.1	2006-01-01	3III_C.2.2	Brazil
**168**	EPI_ISL_19829003	2025-01-13	3III_B.3.2	Brazil	**413**	GU131851.1	2006-01-01	3III_C.2.2	Brazil
**169**	EPI_ISL_19829004	2025-01-19	3III_B.3.2	Brazil	**414**	GU131852.1	2006-01-01	3III_C.2.2	Brazil
**170**	EPI_ISL_19829005	2025-01-12	3III_B.3.2	Brazil	**415**	GU131853.1	2006-01-01	3III_C.2.2	Brazil
**171**	EPI_ISL_19829006	2025-01-20	3III_B.3.2	Brazil	**416**	GU131854.1	2006-01-01	3III_C.2.2	Brazil
**172**	EPI_ISL_19829007	2025-01-22	3III_B.3.2	Brazil	**417**	GU131855.1	2006-01-01	3III_C.2.2	Brazil
**173**	EPI_ISL_19829009	2025-01-13	3III_B.3.2	Brazil	**418**	GU131856.1	2006-01-01	3III_C.2	Brazil
**174**	EPI_ISL_19829010	2025-01-23	3III_B.3.2	Brazil	**419**	GU131857.1	2006-01-01	3III_C.2.2	Brazil
**175**	EPI_ISL_19829011	2025-01-27	3III_B.3.2	Brazil	**420**	GU131858.1	2006-01-01	3III_C.2.2	Brazil
**176**	EPI_ISL_19829012	2025-02-13	3III_B.3.2	Brazil	**421**	GU131859.1	2006-01-01	3III_C.2.2	Brazil
**177**	EPI_ISL_19829013	2025-01-30	3III_B.3.2	Brazil	**422**	GU131860.1	2006-01-01	3III_C.2.2	Brazil
**178**	EPI_ISL_19829014	2025-01-30	3III_B.3.2	Brazil	**423**	GU131861.1	2007-01-01	3III_C.2.2	Brazil
**179**	EPI_ISL_19829015	2025-02-01	3III_B.3.2	Brazil	**424**	GU131862.1	2007-01-01	3III_C.2.2	Brazil

	ID	Collection date	Lineage	Country		ID	Collection date	Lineage	Country
**180**	EPI_ISL_19829016	2024-12-17	3III_B.3.2	Brazil	**425**	GU131865.1	2006-01-01	3III_C.2.2	Brazil
**181**	EPI_ISL_19848681	2025-01-09	3III_B.3.2	Brazil	**426**	GU131866.1	2007-01-01	3III_C.2	Brazil
**182**	EPI_ISL_19894028	2025-03-02	3III_B.3.2	Brazil	**427**	GU131867.1	2007-01-01	3III_C.2.2	Brazil
**183**	EPI_ISL_19894029	2025-03-10	3III_B.3.2	Brazil	**428**	GU131868.1	2007-01-01	3III_C.2	Brazil
**184**	EPI_ISL_19894030	2025-02-26	3III_B.3.2	Brazil	**429**	GU131869.1	2007-01-01	3III_C.2.2	Brazil
**185**	EPI_ISL_19894031	2025-02-19	3III_B.3.2	Brazil	**430**	GU131870.1	2007-01-01	3III_C.2.2	Brazil
**186**	EPI_ISL_19894032	2025-02-19	3III_B.3.2	Brazil	**431**	GU131871.1	2007-01-01	3III_C.2.2	Brazil
**187**	EPI_ISL_19894033	2025-02-19	3III_B.3.2	Brazil	**432**	GU131872.1	2007-01-01	3III_C.2.2	Brazil
**188**	EPI_ISL_19894034	2025-03-12	3III_B.3.2	Brazil	**433**	GU131873.1	2007-01-01	3III_C.2	Brazil
**189**	EPI_ISL_19894035	2025-04-01	3III_B.3.2	Brazil	**434**	GU131874.1	2007-01-01	3III_C.2	Brazil
**190**	EPI_ISL_19894036	2025-04-05	3III_B.3.2	Brazil	**435**	GU131875.1	2007-01-01	3III_C.2.2	Brazil
**191**	EPI_ISL_19895308	2025-01-06	3III_B.3.2	Brazil	**436**	GU131876.1	2007-01-01	3III_C.2.2	Brazil
**192**	EPI_ISL_19895309	2025-01-13	3III_B.3.2	Brazil	**437**	GU131877.1	2007-01-01	3III_C.2	Brazil
**193**	EPI_ISL_19895310	2025-02-11	3III_B.3.2	Brazil	**438**	GU131878.1	2007-01-01	3III_C.2	Brazil
**194**	EPI_ISL_19895311	2025-03-13	3III_B.3.2	Brazil	**439**	JF808118.1	2002-01-01	3III_C.2	Brazil
**195**	EPI_ISL_19895312	2025-03-10	3III_B.3.2	Brazil	**440**	JF808119.1	2004-01-01	3III_C.2	Brazil
**196**	EPI_ISL_19895313	2025-03-17	3III_B.3.2	Brazil	**441**	JF808120.1	2009-01-01	3III_C.2	Brazil
**197**	EPI_ISL_19895314	2025-03-19	3III_B.3.2	Brazil	**442**	JF808121.1	2007-01-01	3III_C.2	Brazil
**198**	EPI_ISL_19895315	2025-03-19	3III_B.3.2	Brazil	**443**	JF808124.1	2003-01-01	3III_C.2	Brazil
**199**	EPI_ISL_19898365	2025-02-12	3III_B.3.2	Brazil	**444**	JF808125.1	2002-01-01	3III_C.2	Brazil
**200**	EPI_ISL_19898366	2025-02-24	3III_B.3.2	Brazil	**445**	JF808126.1	2003-01-01	3III_C.2	Brazil
**201**	EPI_ISL_19898367	2025-03-06	3III_B.3.2	Brazil	**446**	JF808127.1	2002-01-01	3III_C.2	Brazil
**202**	EPI_ISL_19898368	2025-02-27	3III_B.3.2	Brazil	**447**	JN697379.1	2006-01-01	3V	Brazil
**203**	EPI_ISL_19898369	2025-03-08	3III_B.3.2	Brazil	**448**	JX669489.1	2003-01-01	3III_C.2	Brazil
**204**	EPI_ISL_19898370	2025-03-14	3III_B.3.2	Brazil	**449**	JX669490.1	2002-01-01	3III_C.2	Brazil
**205**	EPI_ISL_19898371	2025-03-15	3III_B.3.2	Brazil	**450**	JX669491.1	2002-01-01	3III_C.2	Brazil

	ID	Collection date	Lineage	Country		ID	Collection date	Lineage	Country
**206**	EPI_ISL_19898372	2025-03-13	3III_B.3.2	Brazil	**451**	JX669492.1	2005-01-01	3III_C.2	Brazil
**207**	EPI_ISL_19898373	2025-03-14	3III_B.3.2	Brazil	**452**	JX669493.1	2005-01-01	3III_C.2	Brazil
**208**	EPI_ISL_19898374	2025-03-19	3III_B.3.2	Brazil	**453**	JX669494.1	2005-01-01	3III_C.2	Brazil
**209**	EPI_ISL_19898375	2025-03-21	3III_B.3.2	Brazil	**454**	JX669495.1	2004-01-01	3III_C.2	Brazil
**210**	EPI_ISL_19898376	2025-03-21	3III_B.3.2	Brazil	**455**	JX669496.1	2006-01-01	3III_C.2	Brazil
**211**	EPI_ISL_19898377	2025-03-26	3III_B.3.2	Brazil	**456**	JX669497.1	2005-01-01	3III_C.2	Brazil
**212**	EPI_ISL_19898378	2025-04-04	3III_B.3.2	Brazil	**457**	JX669498.1	2004-01-01	3III_C.2	Brazil
**213**	EPI_ISL_19898379	2025-04-04	3III_B.3.2	Brazil	**458**	JX669499.1	2004-01-01	3III_C.2	Brazil
**214**	EPI_ISL_19898380	2025-04-02	3III_B.3.2	Brazil	**459**	JX669500.1	2005-01-01	3III_C.2	Brazil
**215**	EPI_ISL_19898381	2025-04-02	3III_B.3.2	Brazil	**460**	JX669501.1	2005-01-01	3III_C.2	Brazil
**216**	EPI_ISL_19898382	2025-04-06	3III_B.3.2	Brazil	**461**	JX669502.1	2005-01-01	3III_C.2	Brazil
**217**	EPI_ISL_19898383	2025-04-07	3III_B.3.2	Brazil	**462**	JX669503.1	2005-01-01	3III_C.2	Brazil
**218**	EPI_ISL_19898384	2025-04-07	3III_B.3.2	Brazil	**463**	JX669504.1	2006-01-01	3III_C.2	Brazil
**219**	EPI_ISL_19898385	2025-04-10	3III_B.3.2	Brazil	**464**	JX669505.1	2006-01-01	3III_C.2	Brazil
**220**	EPI_ISL_19898386	2025-03-27	3III_B.3.2	Brazil	**465**	JX669506.1	2006-01-01	3III_C.2	Brazil
**221**	EPI_ISL_19898387	2025-04-15	3III_B.3.2	Brazil	**466**	JX669507.1	2006-01-01	3III_C.2	Brazil
**222**	EPI_ISL_19898388	2025-04-13	3III_B.3.2	Brazil	**467**	JX669508.1	2006-01-01	3III_C.2	Brazil
**223**	EPI_ISL_19898389	2025-04-15	3III_B.3.2	Brazil	**468**	KC425219.1	2002-01-01	3III_C.2	Brazil
**224**	EPI_ISL_19898390	2025-04-08	3III_B.3.2	Brazil	**469**	KF955473.1	2002-01-01	3III_C.2	Brazil
**225**	EPI_ISL_19898391	2025-04-22	3III_B.3.2	Brazil	**470**	OQ706226.1	2023-03-04	3III_B.3.2	Brazil
**226**	EPI_ISL_19898392	2025-04-16	3III_B.3.2	Brazil	**471**	OQ706227.1	2023-01-22	3III_B.3.2	Brazil
**227**	EPI_ISL_19898393	2025-04-16	3III_B.3.2	Brazil	**472**	OQ706228.1	2023-01-03	3III_B.3.2	Brazil
**228**	EPI_ISL_19898394	2025-04-11	3III_B.3.2	Brazil	**473**	OQ727062.1	2006-04-17	3III_C.2	Brazil
**229**	EPI_ISL_19898395	2025-04-22	3III_B.3.2	Brazil	**474**	OQ868517.1	2023-03-12	3III_B.3.2	Brazil
**230**	EPI_ISL_19898396	2025-04-25	3III_B.3.2	Brazil	**475**	PQ882222.1	2023-11-03	3III_B.3.2	Brazil
**231**	EPI_ISL_19898397	2025-04-27	3III_B.3.2	Brazil	**476**	PQ882223.1	2024-01-25	3III_B.3.2	Brazil

	ID	Collection date	Lineage	Country		ID	Collection date	Lineage	Country
**232**	EPI_ISL_19898398	2025-03-27	3III_B.3.2	Brazil	**477**	PQ882224.1	2024-04-20	3III_B.3.2	Brazil
**233**	EPI_ISL_19898399	2025-03-31	3III_B.3.2	Brazil	**478**	PQ882225.1	2024-05-13	3III_B.3.2	Brazil
**234**	EPI_ISL_19898400	2025-03-31	3III_B.3.2	Brazil	**479**	PQ882226.1	2024-05-17	3III_B.3.2	Brazil
**235**	EPI_ISL_19898401	2025-03-26	3III_B.3.2	Brazil	**480**	PQ882227.1	2024-05-22	3III_B.3.2	Brazil
**236**	EPI_ISL_19898402	2025-03-29	3III_B.3.2	Brazil	**481**	PQ882228.1	2024-05-24	3III_B.3.2	Brazil
**237**	EPI_ISL_19898403	2025-03-28	3III_B.3.2	Brazil	**482**	PQ882229.1	2024-05-24	3III_B.3.2	Brazil
**238**	EPI_ISL_19898404	2025-03-28	3III_B.3.2	Brazil	**483**	PQ882230.1	2024-05-29	3III_B.3.2	Brazil
**239**	EPI_ISL_19898405	2025-04-03	3III_B.3.2	Brazil	**484**	PV763902.1	2025-02-06	3III_B.3.2	Brazil
**240**	EPI_ISL_19898406	2025-04-04	3III_B.3.2	Brazil	**485**	PV763903.1	2025-02-07	3III_B.3.2	Brazil
**241**	EPI_ISL_19898407	2025-04-06	3III_B.3.2	Brazil	**486**	PV763904.1	2025-02-03	3III_B.3.2	Brazil
**242**	EPI_ISL_19898408	2025-04-08	3III_B.3.2	Brazil	**487**	PV763905.1	2025-02-06	3III_B.3.2	Brazil
**243**	EPI_ISL_19898409	2025-04-09	3III_B.3.2	Brazil	**488**	PV763906.1	2025-02-25	3III_B.3.2	Brazil
**244**	EPI_ISL_19898410	2025-04-07	3III_B.3.2	Brazil	**489**	PV763907.1	2025-03-22	3III_B.3.2	Brazil
**245**	EPI_ISL_19908994	2025-02-06	3III_B.3.2	Brazil					

Note. IDs in bold correspond to samples generated in this study.

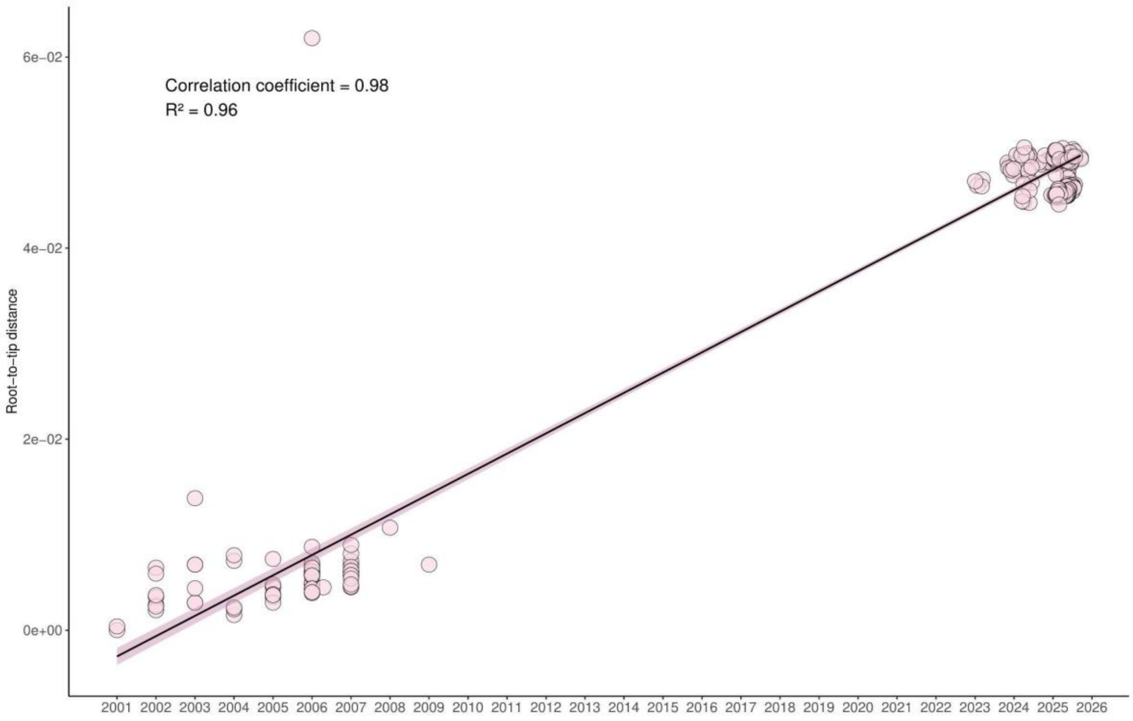

Supplementary Figure S2. Root-to-tip regression analysis of the Brazilian DENV-3 dataset**.** The genetic divergence (root-to-tip distance) is plotted against sampling dates to assess the temporal signal. The strong linear relationship (correlation coefficient = 0.98; R^2 = 0.96) indicates a robust molecular clock signal, supporting the suitability of the dataset for time-scaled phylogenetic and phylodynamic analyses.

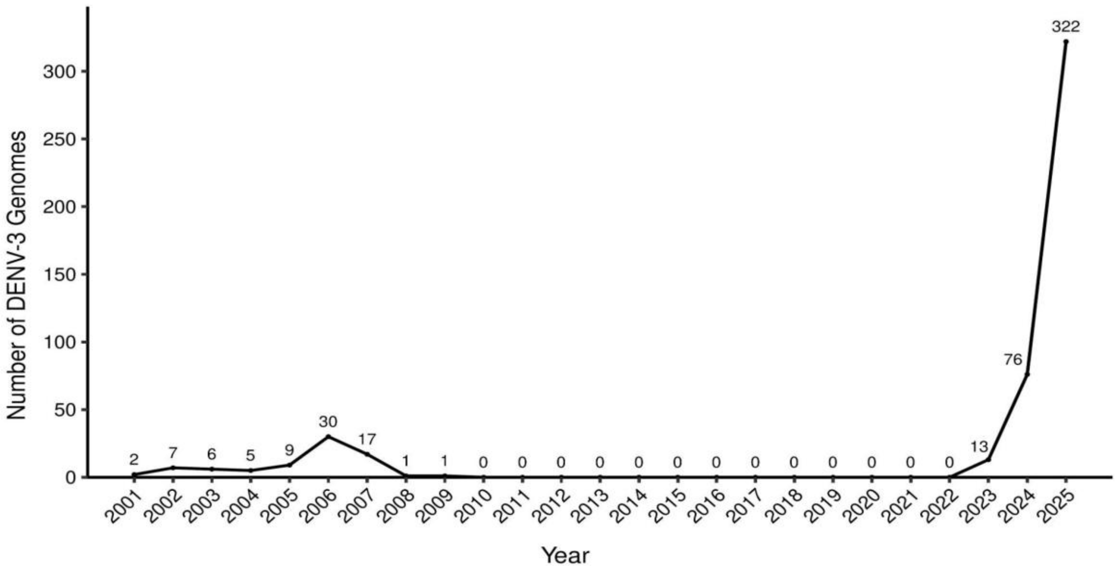

Supplementary Figure S3. Temporal distribution of DENV-3 genomes sequenced in Brazil between 2001 and 2025. The line plot shows the annual number of genomes available, with points

representing the total number for each year. Years with no available genomic data are shown with zero counts, highlighting prolonged periods of limited genomic surveillance.

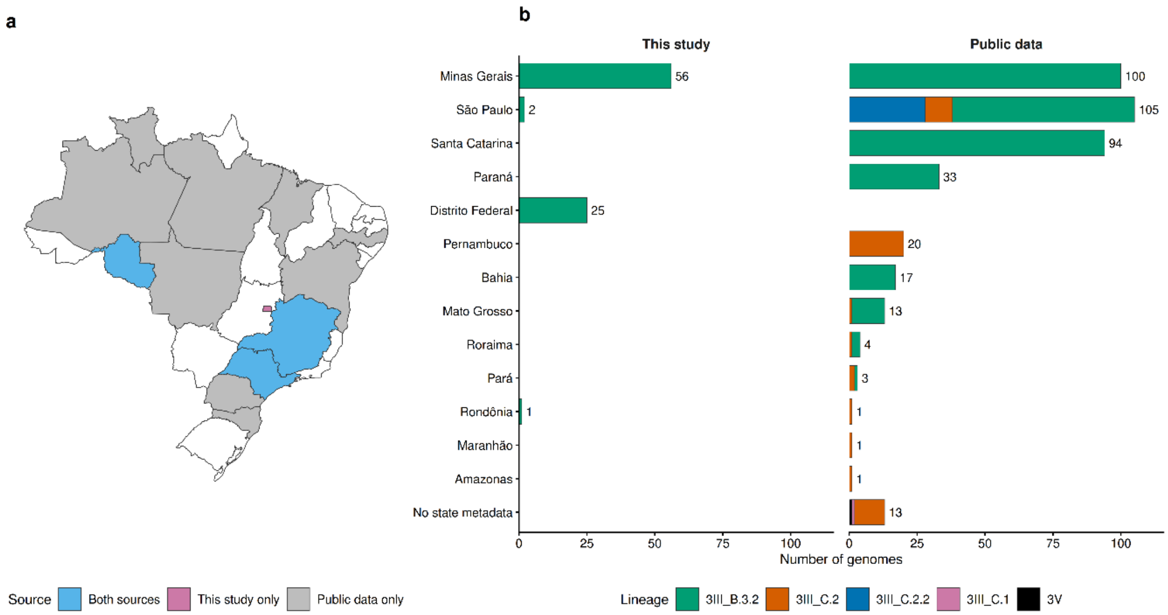

Supplementary Figure S4. Geographic distribution of Brazilian DENV-3 genomes included in this study**. (a)** Brazilian states represented in the genomic dataset according to data source. "Both sources" indicates states with both newly generated genomes and publicly available sequences, "This study only" indicates states represented only by newly generated genomes, and "Public data only" indicates states represented only by sequences retrieved from public databases. **(b)** Number of Brazilian DENV-3 genomes by state, data source, and lineage among sequences included in the dataset. Sequences without state-level metadata are shown separately as "No state metadata" in panel b and were not represented in the map.
